# Dataset of near infrared spectroscopy measurements to predict rheological parameters of sludge

**DOI:** 10.1016/j.dib.2016.09.020

**Published:** 2016-09-21

**Authors:** F. Gibouin, E. Dieudé-Fauvel, J-C. Baudez, R. Bendoula

**Affiliations:** IRSTEA, UMR ITAP, 361 rue Jean-François Breton, BP 5095, 34093 Montpellier, France

**Keywords:** Sludge, Rheological parameters, Near infrared spectroscopy, PLS

## Abstract

In the dataset presented in this article, 36 sludge samples were characterized. Rheological parameters were determined and near infrared spectroscopy measurements were realized. In order to assess the potential of near infrared spectroscopy to predict rheological parameters of sludge, Partial Least Square algorithm was used to build calibration models.

**Specifications Table**

**Table:** 

Subject area	*Physics, Spectroscopy*
More specific subject area	*Wastewater treatment*
Type of data	*Table, figure,.mat file*
How data was acquired	Rheometer (Mars II Thermofisher); Near Infrared Spectrometer (JASCO V-670)
Data format	*Raw, analyzed*
Experimental factors	*36 sludge samples from Middle and South of France were analyzed using a rheometer and Near Infrared Spectrometer coupled with chemometric analysis*
Experimental features	*Near Infrared Spectroscopy coupled with chemometric analysis was used to test the feasibility to predict rheological parameters of sludge samples.*
Data source location	*Middle and South of France*
Data accessibility	*The data is available with this article*

**Value of the data**

• The data can be used as supplements on the physical properties of sludge and can be compared to other studies.
• Those data establish a link between physical properties and reflectance spectra on various sludge samples.
• Near infrared spectroscopy and multivariate analysis are able to predict rheological parameters of sludge.

## 1 Data

Several measurements on 36 sludge samples of different types (primary, secondary, digested, and dehydrated) were made. Rheological parameters (elastic and viscous moduli, yield stress, and viscosity) were determined (Table 1). In parallel, reflectance spectra were measured using an integrating sphere (Fig. 3). With a Partial Least Square (PLS) algorithm, predicting models were obtained for the dry matter (Fig. 4) and four rheological parameters (Fig. 5, Fig. 6, Fig. 7, Fig. 8).

## 2 Experimental design, materials and methods

### 2.1 Sludge sample

36 sludge samples were collected in different wastewater treatment plants in France (Table 1). Consequently, a various panel of samples (primary, secondary, digested, and dehydrated) is available to construct the database. Moreover, knowing that sludges evolve over a large period of time, some samples were measured at different times over a period of 3 months. Additionally, two samples were mixed to create a new sludge. The database is so formed of 36 measurements. Finally, once collected, the samples were stored in sealed cans in the fridge before being characterized.

The dry matter of each sample was determined at 105 °C for 24 h (Table 1).

### 2.2 Rheological measurements

A controlled stress rheometer (Mars II Thermofisher) was used with a coaxial cylinders geometry (*R_in_*=19 mm, *H_in_*=55 mm and *R_out_*=21.5 mm). In addition, both surfaces were rough, which avoids wall slip. The temperature was kept constant (at 20 °C) through a thermostatic bath (C25P Haake).

**Fig. 1:**
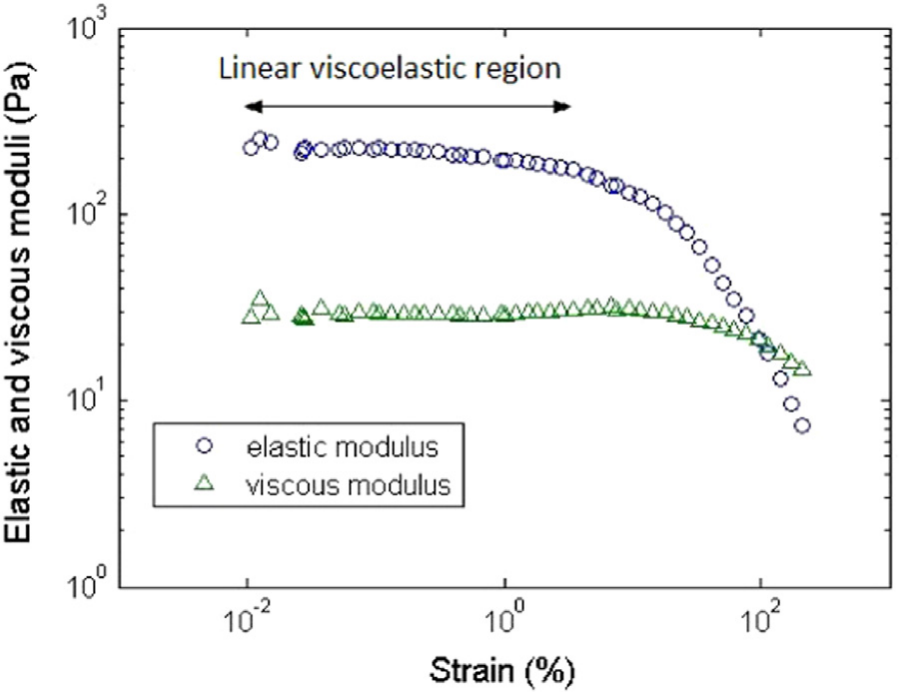
Evolution of the elastic and viscous moduli as a function of the strain for the sample 25.

The procedure consisted in mixing the samples at 300 rpm for 10 min with a blending (RW20 Ika) in order to homogenize them. Then, they were left at rest for 30 min in the measurement geometry in order for the sludge to be restructured. After this rest, viscoelastic properties (Fig. 1) were measured by applying oscillations at a frequency of 1 Hz for a strain range from 0.01% to 200%. Fifty measurement points were recorded according to a logarithmic distribution between those two limits. For each sample, a value of the elastic (*G*’) and the viscous (*G*’’) moduli in the linear viscoelastic region can be extracted (Table 1).

**Fig. 2:**
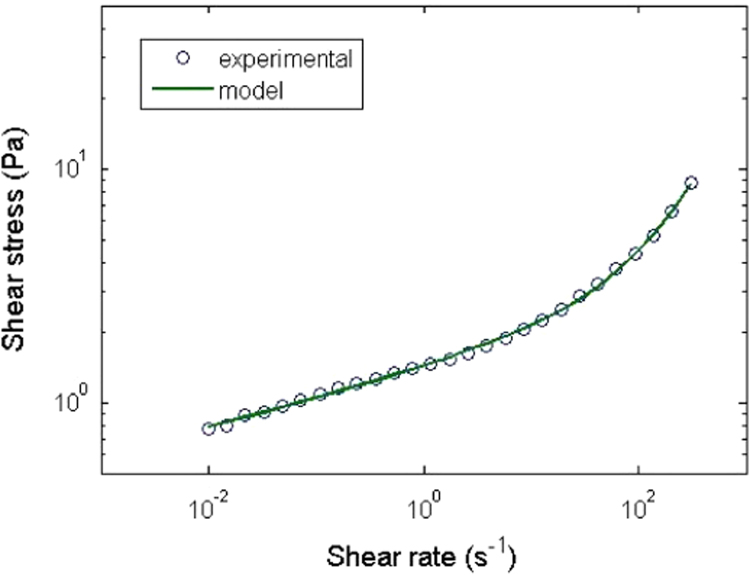
Rheogram of the sample 36 fitted by a modified Herschel–Bulkley model (*τ*_0_=0.207 Pa, *K*=1.226 Pa s^m^, *m*=0.1597, *α*_0_=0.0176 Pa s and *R*^2^=0.99).

Finally, flow properties were obtained by applying a ramp of decreasing shear rates from 1000 s^−1^ to 0.01 s^−1^ (Fig. 2). Thirty measurement points, each for a time of 40 s, were used according to a logarithmic distribution between the two limits. In order to determine the yield stress (*τ*_0_) and the plastic viscosity (*α*_0_) of each sample (Table 1), a modified Herschel–Bulkley model proposed by Baudez et al. [1] was used.

$$\tau =\tau_{0}+K{\dot{\gamma }}^{m}+\alpha_{0}\dot{\gamma }$$

### 2.3 Spectral measurements

All the spectra measurements were realized simultaneously (but separately) with the rheological measurements. The samples had the same history: a mixing at 300 rpm for 10 min and a rest of 30 min. Data were acquired, exported and converted to Matlab readable files.

**Fig. 3:**
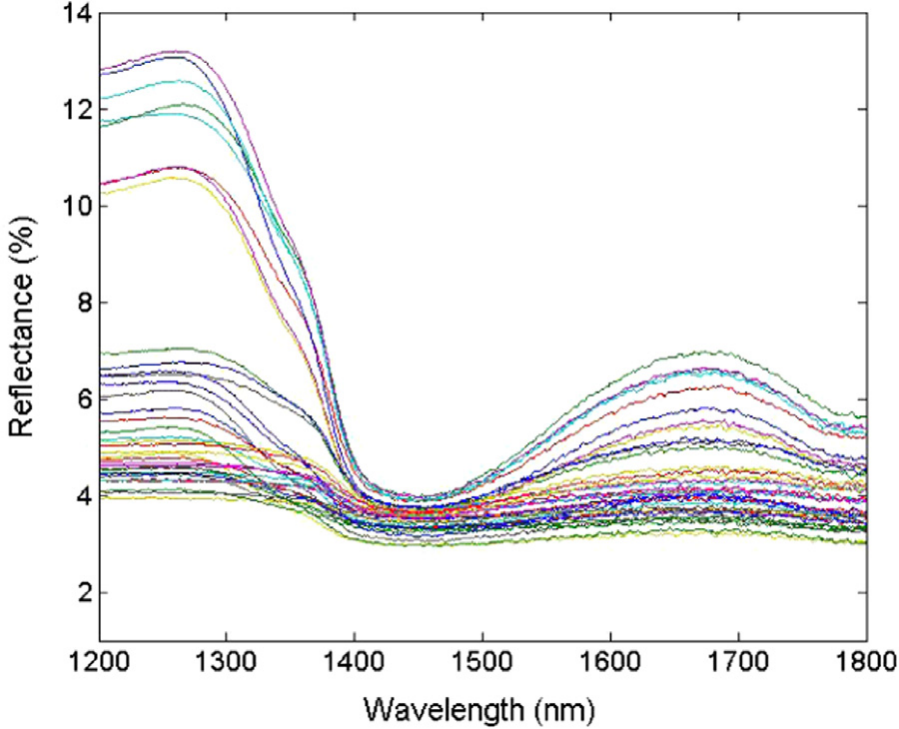
Reflectance spectra measured with an integrating sphere.

Acquisitions were taken with a pre-dispersive spectrometer double beam (JASCO V-670) equipped with an integrating sphere. Samples were analyzed in a quartz cell with optical path of 1 cm (Hellma). Spectral data (Fig. 3) were collected in the wavelength region of 1200–1800 nm at 5 nm intervals and a spectral bandwidth of 12 nm. The baseline was measured with a diffuse reflectance standard (Spectralon@). The manipulation of the experiments was undertaken at controlled room temperature (22±0.5 °C).

### 2.4 PLS algorithm

**Fig. 4:**
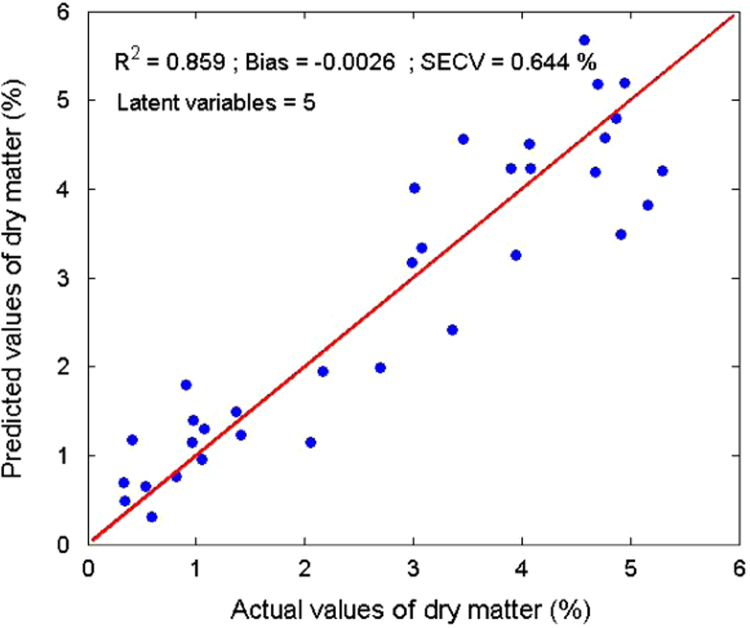
Calibration model for the dry matter.

**Fig. 5:**
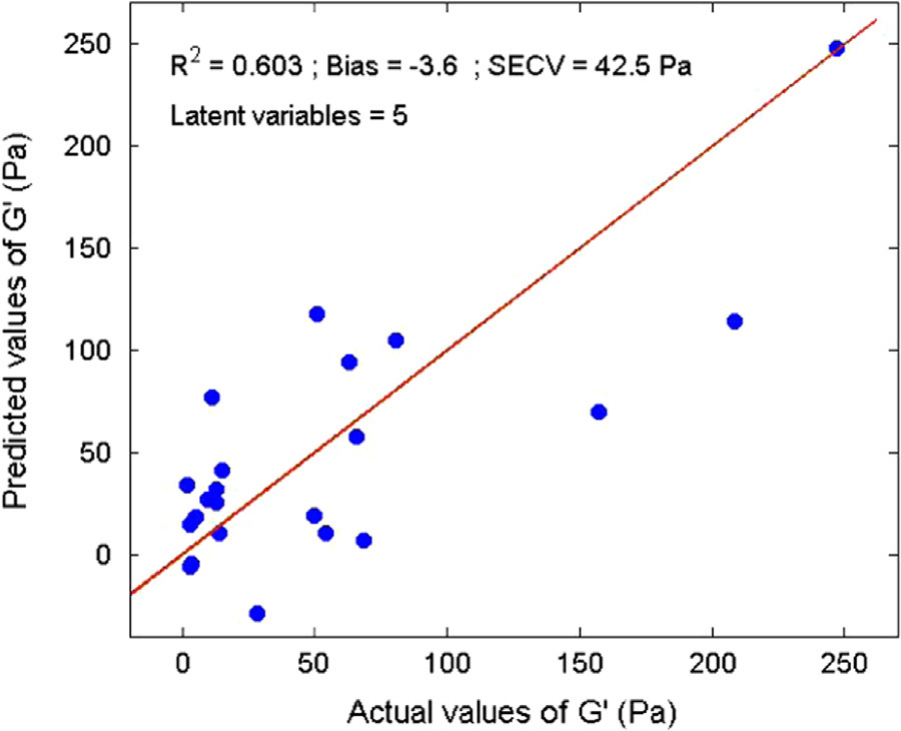
Calibration model for the elastic modulus.

**Fig. 6:**
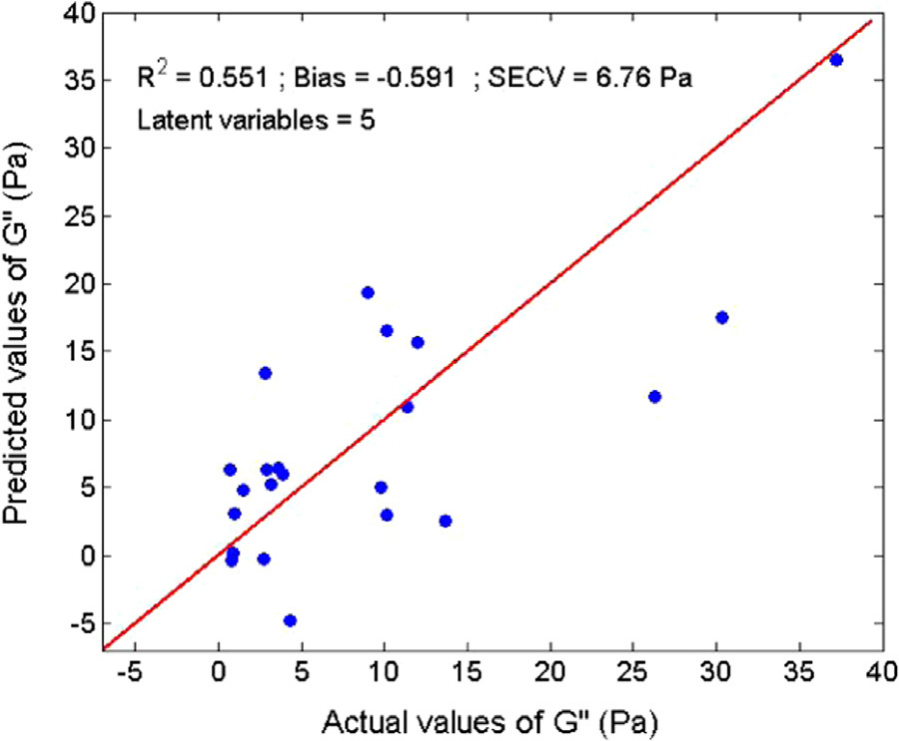
Calibration model for the viscous modulus.

**Fig. 7:**
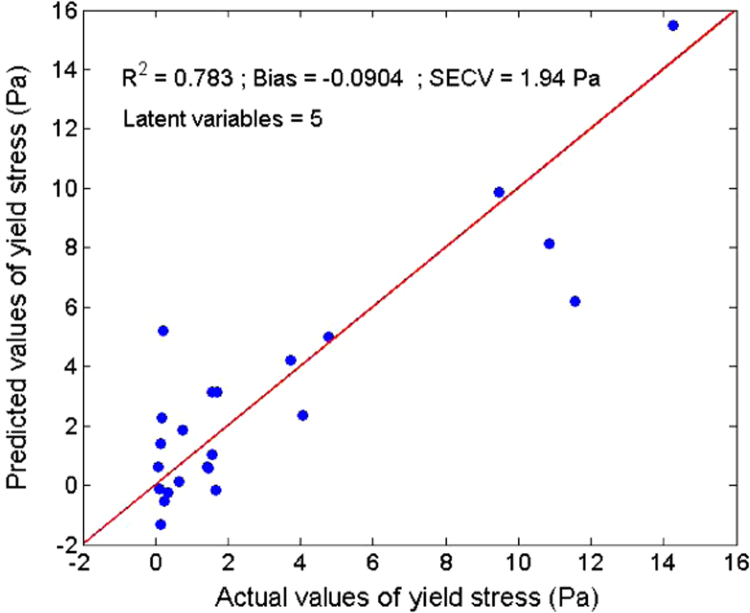
Calibration model for the yield stress.

**Fig. 8:**
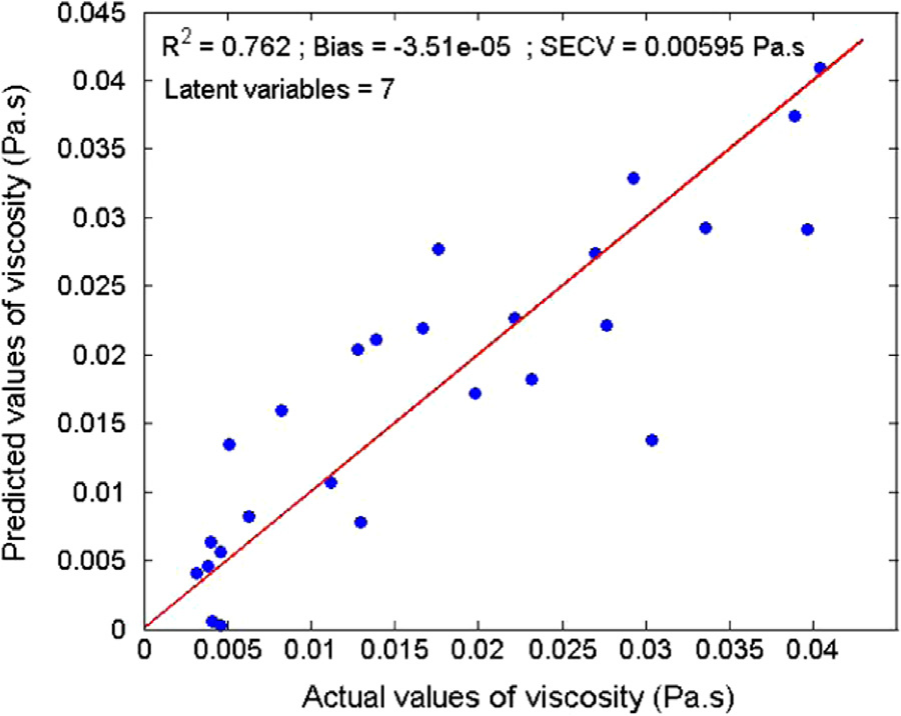
Calibration model for the viscosity.

A Partial Least Square (PLS) [2] algorithm was used to model the physical properties of the sludge. A general PLS model was built using the whole calibration set. The number of latent variables was determined by comparing performances by leave-one-out cross-validation [3]. Model results (Fig. 4, Fig. 5, Fig. 6, Fig. 7, Fig. 8) were evaluated on the basis of the coefficient of determination (*R*²) and the standard error of cross-validation (SECV).

## Figures and Tables

**Table 1:** Location, dry matter and rheological parameters of sludges.

Sample	Wastewater treatment plant	Dry matter (%)	Elastic modulus (Pa)	Viscous modulus (Pa)	Yield stress (Pa)	Viscosity (Pa.s)
1	Castries	1.408	13.969	2.763		
2	Lyon	3.016				
3	Lyon	4.076	50.644	8.977		
4	Lyon		247.215	37.226	14.250	0.0404
5	Moulins sur Allier		12.922	3.147	1.559	0.0128
6	Vichy	0.592				
7	Vichy	1.074				0.0038
8	Vichy	3.895	80.505	10.127	3.739	0.0167
9	Varennes sur Allier	4.943	157.259	26.267	11.550	0.0389
10	Castries	0.958			0.144	0.0040
11	Castries	1.368	3.245	0.905	0.337	0.0063
12	Lyon	4.874			9.480	0.0293
13	Moulins sur Allier	3.362	14.965	3.598	1.692	0.0198
14	Varennes sur Allier	0.331				
15	Moulins sur Allier	0.978				0.0031
16	Varennes sur Allier	0.530				
17	Moulins sur Allier	0.905				
18	Montpellier	5.293	49.505	9.782	1.463	0.0276
19	Montpellier	3.076	5.068	1.484	0.074	0.0082
20	Montpellier	2.692				
21	Baillargues Saint Brès	0.414				
22	Baillargues Saint Brès	1.052	2.662	0.774	0.262	0.0046
23	Baillargues Saint Brès	0.338				
24	Montpellier	4.912	68.527	13.641	1.674	0.0303
25	Lyon	4.767	208.299	30.325	10.840	0.0396
26	Lyon + Montpellier	4.681	12.703	3.903	1.413	0.0232
27	Lyon + Montpellier	4.695	65.756	11.362	4.070	0.0270
28	Lyon + Montpellier	4.579	62.975	11.988	4.770	0.0336
29	Castries	2.049	28.423	4.288	1.549	0.0130
30	Castries	0.815				0.0041
31	Montpellier	3.464			0.174	0.0139
32	Montpellier	3.944	54.191	10.123	0.667	0.0112
33	Montpellier	2.994	1.678	0.758	0.130	0.0051
34	Montpellier	4.066	9.659	2.902	0.762	0.0222
35	Montpellier	2.172	2.803	1.008	0.122	0.0046
36	St Germain des Fossés	5.164	11.393	2.796	0.207	0.0176
